# Inflammatory profile of vertically HIV-1 infected adolescents receiving ART in Cameroon: a contribution toward optimal pediatric HIV control strategies

**DOI:** 10.3389/fimmu.2023.1239877

**Published:** 2023-08-14

**Authors:** Aude Christelle Ka’e, Aubin Joseph Nanfack, Georgia Ambada, Maria Mercedes Santoro, Desire Takou, Ezechiel Ngoufack Jagni Semengue, Alex Durand Nka, Marie Laure Mpouel Bala, Orphelie Ndoh Endougou, Elise Elong, Grace Beloumou, Sandrine Djupsa, Davy Hyacinthe Gouissi, Nadine Fainguem, Michel Carlos Tommo Tchouaket, Samuel Martin Sosso, Daniel Kesseng, Francis Ateba Ndongo, Nelson Sonela, Arnaud Cedric Lacmago Kamta, Hyppolite K. Tchidjou, Therese Ndomgue, Suzie Tetang Moyo Ndiang, Anne Esther Njom Nlend, Celine Nguefeu Nkenfou, Carla Montesano, Gregory Edie Halle-Ekane, Giulia Cappelli, Caroline T. Tiemessen, Vittorio Colizzi, Francesca Ceccherini-Silberstein, Carlo-Federico Perno, Joseph Fokam

**Affiliations:** ^1^ Chantal BIYA International Reference Centre for Research on HIV/AIDS Prevention and Management (CIRCB), Yaounde, Cameroon; ^2^ Department of Experimental Medicine, University of Rome Tor Vergata, Rome, Italy; ^3^ Faculty of Science, University of Yaounde 1, Yaounde, Cameroon; ^4^ Faculty of Medicine and Biomedical Sciences, University of Yaoundé 1, Yaoundé, Cameroon; ^5^ School of Health Sciences, Catholic University of Central Africa, Yaounde, Cameroon; ^6^ Mother and Child Centre, Chantal BIYA Foundation, Yaounde, Cameroon; ^7^ Division of Operational Health Research, Ministry of Public Health, Yaounde, Cameroon; ^8^ Faculty of Medicine and Biomedical Sciences, University of Garoua, Garoua, Cameroon; ^9^ Elisabeth Glaser Pediatric AIDS Foundation (EGPAF), Country-office, Yaoundé, Cameroon; ^10^ HIV Management Unit, Mfou District Hospital, Mfou, Cameroon; ^11^ Amiens University Hospital, Amiens, France; ^12^ National Social Welfare Hospital, Yaounde, Cameroon; ^13^ Higher Institute of Medical Technology, Yaounde, Cameroon; ^14^ Faculty of Health Sciences, University of Buea, Buea, Cameroon; ^15^ National Research Council, Rome, Italy; ^16^ National Institute for Communicable Diseases and Faculty of Health Sciences, University of the Witwatersrand, Johannesburg, South Africa; ^17^ Faculty of Science and Technology, Evangelic University of Cameroon, Bandjoun, Cameroon; ^18^ Bambino Gesu Pediactric Hospital, Rome, Italy

**Keywords:** HIV-1, inflammation, antiretroviral therapy, adolescents, cytokines, viral load, CD4

## Abstract

Antiretroviral therapy (ART) has improved the lifespan of people living with HIV. However, their immune system remains in a state of sustained activation/inflammation, which favors viral replication and depletion of helper T-cells with varying profiles according to ART-response. We herein sought to ascertain the inflammatory profile of adolescents living with perinatal HIV-1 infection (ALPHI) receiving ART in an African context. In this cross-sectional and comparative study among ART-experienced ALPHI in Yaoundé-Cameroon, HIV-1 RNA was measured by Abbott Real-time PCR; CD4 cells were enumerated using flow cytometry; serum cytokines were measured by ELISA; HIV-1 proviral DNA was genotyped by Sanger-sequencing; and archived drug resistance mutations (ADRMs) were interpreted using Stanford HIVdb.v9.0.1. Overall, 73 adolescents were enrolled (60 ALPHI and 13 HIV-1 negative peers) aged 15 (13-18) years; 60.00% were female. ART median duration was 92 (46-123) months; median viral load was 3.99 (3.17-4.66) RNA Log_10_ (copies)/mL and median CD4 count was 326 (201-654) cells/mm^3^. As compared to HIV-negative adolescents, TNFα was highly expressed among ALPHI (p<0.01). Following a virological response, inflammatory cytokines (IFNγ and IL-12), anti-inflammatory cytokines (IL-4 and IL-10) and inflammation-related cytokines (IL-6 and IL-1β) were highly expressed with viral suppression (VS) vs. virological failure (VF), while the chemokine CCL3 was highly expressed with VF (p<0.01). Regarding the immune response, the inflammatory cytokine TNFα was highly expressed in those that are immunocompetent (CD4≥500 cell/mm^3^) vs. immunocompromised (CD4<500 cell/mm^3^), p ≤ 0.01; while chemokine CCL2 was highly expressed in the immunocompromised (p<0.05). In the presence of ADRMs, IL-4 and CCL3 were highly expressed (p=0.027 and p=0.043 respectively). Among ART-experienced ALPHI in Cameroon, the TNFα cytokine was found to be an inflammatory marker of HIV infection; IFNγ, IL-1β, IL-6, and IL-12 are potential immunological markers of VS and targeting these cytokines in addition to antiretroviral drugs may improve management. Moreover, CCL3 and CCL2 are possible predictors of VF and/or being immunocompromised and could serve as surrogates of poor ART response.

## Introduction

Children and adolescents constitute a growing population of people living with HIV in developing countries, mainly due to ongoing mother to child transmission of HIV-1 (MTCT) and the benefits of antiretroviral therapy (ART) in sustaining the lifespan of infected children (1). Of note, sub-Saharan Africa (SSA) is paying the heaviest toll of pediatric HIV as ~85% of worldwide new pediatric infections occur in this region (2). Even though ART has substantially contributed to reducing HIV-1 MTCT (3), women in SSA settings (Cameroon included) (4) still face delayed HIV-1 diagnosis during pregnancy/breastfeeding, which leads to limited coverage in prevention of MTCT (PMTCT) and occurrence of new pediatric HIV-1 infections (5),

With more than 160,000 new cases of HIV-infection reported among children in 2021, pediatric HIV remains a major public health concern, thus calling for novel strategies to ensure optimal management of such a lifelong condition from childhood (1, 2). As in HIV-infected adults, ART increases the life expectancy of HIV-infected infants and children, allowing them to grow toward adulthood. However, the differential features in HIV pathogenesis between adults and children (i.e., faster disease progression and lower control of viral replication) require innovative approaches to optimize the outcomes with current pediatric ART in this underserved population (6–9). Moreover, the inability of antiretrovirals to access latent reservoirs, characterized by infected long-lived memory CD4^+^ T lymphocytes and macrophages, results in the persistent immune activation and inflammation observed in the course of HIV-1 infection (6–8).

Several cytokines have been associated with HIV-1 infection and pathogenesis (10–14). Of note, inflammatory cytokines, including interleukins (IL)-2, IL-6, IL-12, interferon gamma (IFNγ), and tumor necrosis factor-α (TNFα), were reported to be essential for the clearance of HIV infection (10). Furthermore, IL-4, IL-5, IL-10, and IL-13 are known to be associated with disease progression (13, 15). However, such knowledge gaps still exist in the frame of pediatric HIV-infection, and specifically within sub-Saharan Africa (SSA) clinical settings.

An effective immune response against HIV must strike a balance between inflammatory and anti-inflammatory cytokines. Even though ART acts by trying to restore this equilibrium, it is still unclear how immunologic and virologic parameters of adolescents living with perinatal HIV-1 infection (ALPHI) influence circulating cytokine levels. In view of optimizing immunotherapeutic strategies toward HIV pediatric control, the objective of this study was to determine the cytokine profile of ALPHI in Cameroon and to assess the relationship between cytokine levels and the virologic or immunologic responses.

## Materials and methods

### Study design

A cross-sectional observational and comparative study was conducted among 73 adolescents (60 HIV-positive and 13 HIV-negative, ratio 5:1) aged 13-18 years at the Chantal BIYA International Reference Centre for research on HIV/AIDS prevention and management (CIRCB) in Yaoundé-Cameroon for the period ranging from March to July 2019.

Briefly, 60 ART-experienced ALPHI (60.00% were female) were enrolled from reference pediatric health facilities in the city of Yaoundé-Cameroon (Essos Health Centre and the Mother-Child Centre of the Chantal BIYA Foundation), as previously described within the frame of the EDCTP READY-Study cohort (16). Additionally, 13 age-matched HIV-negative adolescents, without malaria and hepatitis B/C infections, were enrolled as a control group.

### Sampling and data collection

From each participating ALPHI, the following parameters were collected: date of HIV diagnosis, age, gender, date of ART initiation, and complete ART history (duration/regimen). Then, 8 mL of whole blood was collected in EDTA tubes for immunological (CD4 count and cytokine testing) and virological (HIV-1 viral load, HIV-1 proviral DNA amplification, and sequencing) analyses.

### HIV-1 RNA measurement

A reverse transcription-polymerase chain reaction (RT-PCR) assay was performed on 700µL of plasma samples using the Abbott RealTime (Abbott Park, IL, USA), an in vitro platform with a lower and upper detection limit of 40 and 10,000,000 copies/mL respectively. RT-PCR was then performed to quantify the HIV-1 viral RNA (plasma viral load, PVL) of each participant by RNA extraction from plasma followed by reverse-transcription/amplification of RNA extracts and simultaneous revelation as per the manufacturer’s instructions (www.molecular.abbott/int/en/products/infectious-disease/realtime-hiv-1-viral-load). For the purpose of the study, the PVL results of each ALPHI were classified as either viral suppression (PVL<1000 copies/mL) or virological failure (PVL≥1,000 copies/mL).

### Helper CD4+ T cells count

CD4+ T cells were enumerated by flow cytometry with a counter cytometer, using the CD4 easy count kit as per the manufacturer’s instructions (Sysmex Partec GmbH) as previously described (17). For the purpose of the study, ALHPI were then classified as either immunocompetent (CD4 ≥ 500 cells/µL) or immunocompromised (CD4 < 500 cells/µL).

### Cytokine measurement

Inflammatory (IL-1β, IL-6, IL-12, CCL2, CCL3, CCL4, IFNγ, IL-17A, and TNFα), anti-inflammatory (IL-4 and IL-10), and inflammation-related cytokine (IL-6 and TGFβ1) measurement was performed on plasma using the sandwich-based enzyme-linked immunosorbent assay (ELISA) with Multi-Analyte ELISArray (Qiagen, Frederick, Maryland, 21704, USA) following the manufacturer’s recommendations (https://www.qiagen.com/us/products/discovery-and-translational research/functional-and-cell-analysis/elisa-assays/multi-analyte-elisarray-kits) (18). The optical density was measured at 450nm (OD_450_), and the concentration in pg/mL was calculated based on the logarithmic function y=a ln(x)+b obtained through the standard curves of samples with known concentrations. OD_450_ reads between 0 and 2.5 were considered to be within the linear range for all of the analytes as instructed (https://www.qiagen.com/dk/resources/faq?id=c9815b21-393e-4cef-8968-5d4e67801aa1&lang=en).

### HIV-1 DNA extraction and genotyping

HIV-1 DNA extraction was done from 200 μL of buffy coat following the protocol of the QIAamp DNA Mini Kit (Qiagen, Maryland, USA). Viral DNA extracts were directly amplified using a two-round PCR according to an in-house genotyping protocol as previously described (9). Sanger-sequencing was performed by capillary electrophoresis on ABI 3500 (Applied Biosystems) using the dye deoxy-termination chain with a set of eight sequencing primers. HIV-1 sequences were assembled and edited using Recall v.2.28 software. Edited sequences were then analyzed for the interpretation of HIV-1 archived drug resistance mutations (ADRMs) using the Stanford HIV Drug Resistance Database algorithm v.9.0.1 (https://hivdb.stanford.edu/). Sequences with more than three mutations in the reverse transcriptase (RT) and/or two mutations in the protease (PR) known to be associated with APOBEC3G/F activity were excluded due to event of hypermutation (19).

### Statistical and phylogenetic analysis

Data were coded and recorded in an excel spreadsheet, cleaned, and double checked. The parameters of central tendency (median) and dispersion (interquartile range) were used to describe discontinuous variables. Categorical variables were described in terms of proportions and frequencies. A Mann-Whitney test was used to compare the mean of continuous variables between the following groups: i) ALPHI vs. uninfected adolescents; ii) ALPHI on viral suppression vs. virological failure; iii) immune-competent vs. immunocompromised ALPHI; iv) ALPHI with vs. without ADRMs. A Spearman correlation test was performed between the PVL/CD4 T cell and each cytokine. Any p-value<0.05 was considered statistically significant.

### Ethical and regulatory considerations

As per the Declaration of Helsinki adopted by the 18^th^ World Medical Assembly in 1964 with respect to international regulations for ethics and good clinical practices, ethical clearance for the present study was obtained from the National Ethics Committee for research on human health (reference N° 2021/12/CE/CNERSH/SP). Administrative authorisations were obtained from the Directorate General of CIRCB and the Directors of clinical sites. Confidentiality was ensured by the use of de-identified and anonymised datasets. Written informed consent was obtained from parents or legal guardians, and written assent was provided by each participating adolescent. For the purpose of beneficence, all laboratory results (HBV, Malaria, CD4, PVL, genotypic drug resistance testing) related to the clinical management of the study participants were provided free of charge for personalized case management toward optimal HIV treatment or treatment of any reported co-infection or comorbidity.

## Results

### Socio-demographic, immuno-virological, and treatment characteristics of HIV-1 infected adolescents

Out of the 60 ALPHI, 36 (60%) were female, and the median [interquartile range, IQR] age was 15 [13-18] years. All were receiving ART and the median [IQR] duration on treatment was 92 [46-123] months; 80% were on non-nucleoside reverse transcriptase inhibitor (NNRTI)-based first-line regimens. Regarding the WHO clinical staging, the majority (70%) were categorized as clinical stage I, and 16.7% were categorized as clinical stage II. Regarding virological response, the median PVL was 3.99 [3.17-4.66] log_10_(copies/mL), of whom about three quarters (n=47) of participating ALPHI were experiencing virological failure (VL≥1000 copies/mL) as defined by WHO in our context. See detailed description in **Table 1**.

**Table 1:** Socio-demographic, immuno-virological, clinical, and therapeutic features of ALPHI.

Gender	Female, n (%)	36 (60.00)
	Male, n (%)	24 (40.00)
Age at enrollment, years	Median [IQR]	15 [13-18]
ART duration, months	Median [IQR]	92 [46-123]
	<60 months, n (%)	17 (28.33)
	≥60 months, n (%)	36 (60.00)
	Unknown, n (%)	7 (11.67)
ART exposition	First-line (NNRTI based regimen)	48 (80.00)
	Second line (PI based regimen)	12 (20.00)
WHO Clinical stages	I, n (%)	42 (70.00)
	II, n (%)	10 (16.67)
	III, n (%)	6 (10.00)
	IV, n (%)	2 (3.33)
	Median [IQR] (log_10_(copies/mL))	3.99 [3.17-4.66]
Viral load, copies/mL	<1000, n (%)	13 (21.67)
	≥1000, n (%)	47 (78.33)
CD4+ T cells	Median [IQR]	326 [201-654]
	<500, n (%)	20 (62.5%)
	≥500, n (%)	12 (37.5%)

ART, Antiretroviral therapy; NNRTI, non-nucleoside reverse-transcriptase inhibitors; PI/r, ritonavir-boosted protease inhibitor; CD4, cluster of differentiation.

### Profile of cytokines and HIV-1 status

Out of all cytokines measured, only the concentration of the inflammatory cytokine TNFα was significantly higher in HIV-1 infected participants compared to their HIV-negative peers (p<0.01), indicating its role as a marker of inflammation or immune activation (**Table 2**).

**Table 2:** Profile of cytokines according to HIV-1 status of adolescents.

Cytokines/ Chemokines	Distribution according to HIV-1 status		*p*-value
	Negative (13) (Median [IQR] in pg/mL)	Positive (60) (Median [IQR] in pg/mL)	
IL-1β	13.8 [11.1-16.6]	10.0 [10.0 – 10.3]	0.26
IL-4	10.1 [10.0-10.2]	10.1 [10.0-10.8]	0.21
IL-6	13.9 [11.1-19.0]	10.5 [10.0-11.8]	0.08
IL-10	10.6 [10.0-12.0]	11.3 [10.0-14.0]	0.39
IL-12	13.1 [10.0-42.0]	10.2 [10.0-11.7]	0.20
IL-17A	14.8 [10.4– 19.6]	13.2 [11.3-18.0]	0.49
IFNγ	10.6 [10.0-11.0]	11.2 [10.2-14.9]	0.42
**TNFα**	**16.8 [14.5-18.0]**	**78.3 [55.1-112.1]**	**<0.01**
TGF-β1	234.7 [10.0-240.5]	225.4 [203.25-258.8]	0.09
CCL2	11.5 [10.0-22.4]	12.9 [10.1-90.1]	0.97
CCL3	18.1 [15.2-39.5]	10.9 [10.0-15.4]	0.24
CCL4	15.4 [10.6-20.0]	10.0 [10.0-12.1]	0.19

IL, interleukin; TGF, transformative growth factor; TNF, tumor necrosis factor.

Bold: statistical significant.

### Profile of cytokines and virological response

**Figure 1:**
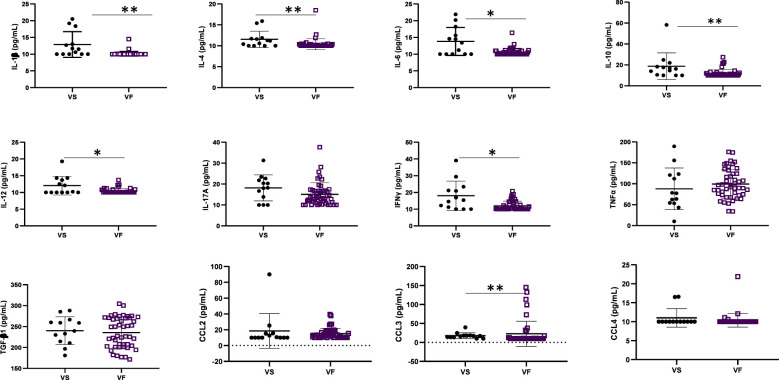
Cytokine profile according to virological response. VF, Virological failure (viral load ≥3 Log_10_(copies/mL)); VS, Viral suppression (viral load <3 Log_10_(copies/mL)); IL, interleukin; TGF, transformative growth factor; TNF, tumor necrosis factor; (p<0.05), ** (p<0.01). This graph shows the significant low level of inflammatory cytokines IL-12 and IFNy; anti-inflammatory cytokines IL-4 and IL-10 and the inflammation related cytokines IL-1β, IL-6 while the level of chemokine CCL3 was high in adolescents with VF as compared to those with VS.

**Figure 2:**
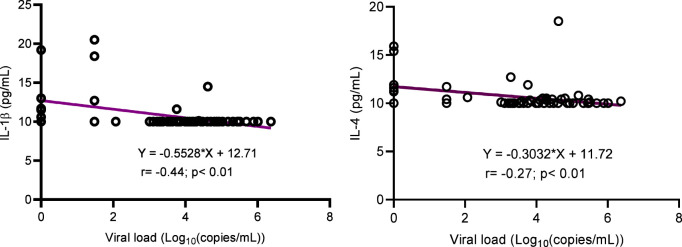
Correlation between cytokines (IL-1β and IL-4) and viral load. IL, interleukin. This graph shows the negative correlation between cytokines IL-1β/IL-4 and the plasmatic viral load highlighting the decrease of such interleukins with increasing levels of viral load.

The concentration of inflammatory cytokines IL-1β, IL-12, and IFNγ; anti-inflammatory cytokine IL-4; and inflammation-related cytokine IL-6 were significantly higher in ALPHI with viral suppression compared to those with virological failure; *p*<0.05 (**Figure 1**). Additionally, the anti-inflammatory cytokine IL-10 was also highly expressed among ALPHI experiencing viral suppression, while the chemokine CCL3 was significantly expressed among ALPHI experiencing virological failure as compared to those on viral suppression; *p*<0.01 (**Figure 1**) However, only cytokines IL-1β and IL-4 were negatively correlated with PVL (r= -0.44; p<0.01 and r= -0.27; p=0.04 respectively; **Figure 2**), suggesting that lower levels of IL-1β and IL-4 are associated with increased severity of PVL, while chemokine CCL2 alone was positively correlated with PVL; r= 0.36; p<0.01.

### Profile of cytokines and immune response

According to the distribution of CD4 cells count in the study population, the median value [IQR] was 326 [201-654] cells/mm^3^, of which 20 (62.5%) participants were immunocompromised (CD4<500 cells/mm^3^) and 12 (37.5%) were immunocompetent (CD4≥500 cells/mm^3^) as per the classification of the World Health Organization (**Table 1**).

**Figure 3:**
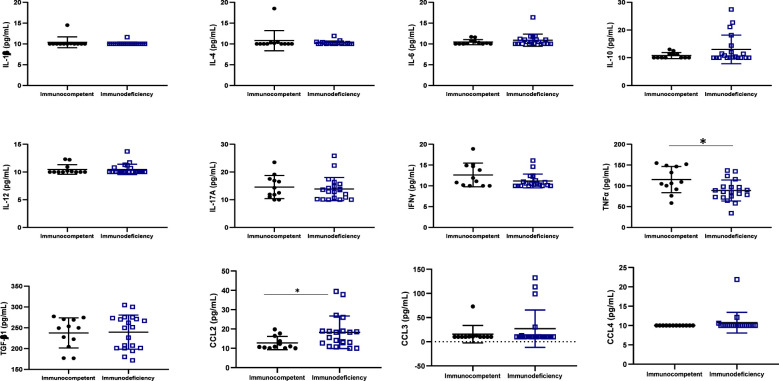
Cytokine profile according to immunocompetence. IL, interleukin; TGF, transformative growth factor; TNF, tumor necrosis factor; Immunocompetent: CD4≥500 cells/mm^3^; immunodeficiency: CD4<500 cells/mm^3^; * (p<0.05); N=32. This graph shows the significant high level of the inflammatory cytokine TNFα in immunocompetent adolescents as compared to those with immunodeficiency while, the level of the chemokine CCL2 was high in adolescents with immunodeficiency as compared to those with good immune response.

One inflammatory cytokine (TNFα) was highly expressed in immunocompetent ALPHI (p ≤ 0.01), while one anti-inflammatory chemokine CCL2 was highly expressed in immunocompromised ALPHI (*p*<0.05), as shown in **Figure 3**. Interestingly, both biomarkers (TNFα and CCL2) correlated significantly with the CD4 T cell count (r= 0.42, p=0.01 and r= -0.43, p=0.01 respectively), thus confirming their clinical significance in pediatric HIV infection.

### Profile of cytokines and antiretroviral therapy regimen

No significant association was found between the ART regimen (NNRTI-based versus PI/r) and the profile of studied cytokines/chemokines (data not shown), suggesting minimal (if any) effect of the ART regimen on the profiling and dynamics of cytokines/chemokines during the course of HIV pediatric infection.

### Profile of cytokines, HIV-1 clades, and archived drug resistance mutations

Out of 32 adolescents from whom HIV-1 genotyping was successful in proviral DNA, 22 (68.75%) were infected with CRF02_AG, 04 (12.50%) with subtype G, 01 (3.13%) with each of the following: recombinant A1/G/K, A1/G, CRF11_cpx, CRF13_cpx, CRF18_cpx, CRF37_cpx, and subtype H. There was no preferential pattern in cytokine profiling according to HIV-1 clade distribution (data not shown).

**Figure 4:**
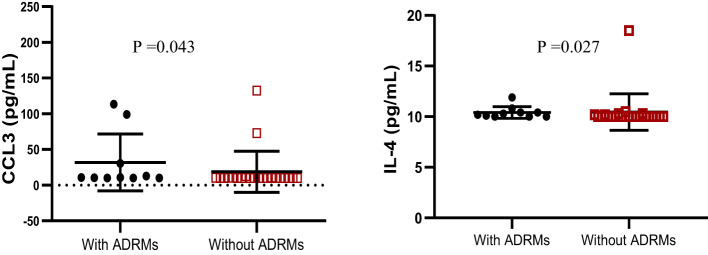
Profile of IL-4 and CCL3 according to the occurrence of ADRMs. ADRMs, Archived Drug Resistance Mutations; IL,interleukin. This graph shows the significant high level of the chemokine CCL3 as well as the cytokine IL-4 among adolescents with ADRMs.

According to HIV-1 mutational profile in proviral DNA, 10 participants (31.25%) harbored archived drug resistance mutations (ARDMs), including 10/32 (31.25%) with NNRTI resistance-associated mutations, 8 (25.00%) with NRTI resistance-associated mutations, and only 1 (3.12%) with a ritonavir-boosted protease inhibitor (PI/r) resistance-associated mutation. The profile of cytokines according to the presence of ADRMs revealed that the levels of Il-4 cytokines and CCL3 were significantly higher in adolescents with ADRMs; p<0.05 (**Figure 4**). The other cytokines/chemokines (IL-1β, IL-6, IL-10, IL-12, MCP1, CCL4, IFNγ, IL-17A, TNFα, and TGF-β1) were not significantly associated to the presence of ADRMs (p=0.19, p= 0.85, p=0.44, p=0.57, p=0.92, p=0.14, p=0.11, p=0.71, p=0.33, p=0.81 respectively).

## Discussion

With the goal to contribute to the control of pediatric HIV-infection in the era of ART and to further support the UNAIDS agenda for research into a functional HIV cure in SSA, the present study has provided new insights on the expression of inflammatory (IL-1β, IL-6, IL-12, CCL2, CCL3, CCL4, IFNγ, IL-17A and TNFα), anti-inflammatory (IL-4 and IL-10), and inflammation-related (Il-6 and TGF-β1) cytokines/chemokines in APHI in a typical SSA setting like Cameroon. Taking into consideration the ART paradigm and the high burden of pediatric HIV-infection in SSA settings like Cameroon (4, 9), this setting represents an ideal avenue for setting up baseline investigations that could pave the way for pediatric HIV cure research in this sub-region.

Among participants, the gender distribution was similar among APHI, suggesting an even distribution of the studied parameters. Moreover, the majority (4/5) of APHI were on an NNRTI-first-line ART regimen (in accordance with the proportion of national ART regimens at the time of the study implementation), and a majority (3/4) were also on a virological failure regimen. Despite the fact that the observed rate of virological response was primarily related to sampling for the purpose of our study objectives, poor ART responses among these APHI has been previously reported, driven by poor adherence and the low-genetic barrier to HIV resistance of first-generation NNRTIs that were commonly used, hence supporting transition to newer regimens (20, 21).

Regarding the profile of inflammatory cytokines, only TNFα was highly expressed among HIV-1-infected adolescents compared to their HIV-negative peers. This underscores the fact that TNFα is highly produced during HIV infection to increase antiviral immunity (22, 23) but also to induce NF-kB, which in turn drives proviral transcription and HIV replication (24). Although previous studies have indicated that TNF-α stimulates HIV-1 replication in cultured PBMC (25, 26), recent studies have found no correlation between levels of TNF-α and HIV-1 replication in lymphoid tissue (26). Further, one group has demonstrated that TNF-α suppresses HIV-1 production in peripheral blood monocytes (PBM) (27, 28). These studies indicate that TNF-α may have distinct effects on the pathogenesis of HIV.

Regarding virological response, our findings underscore a decrease in inflammation-related cytokines Il-1β and the anti-inflammatory cytokine IL-4 with increasing levels of plasmatic viral load being observed. Although during the course of HIV-1 infection the level of Il-1β is increased in different anatomical compartments, particularly in lymphatic tissues, and this elevation is associated with disease progression off-ART (29), a consequence of immune depletion and chronic immune activation following HIV replication could be the progressive decrease of the cytokines over time with antiretroviral treatment. On the other hand, HIV can directly infect and deplete specific immune cells that are responsible for producing IL-4, further contributing to its reduced levels (30) as observed in Brazilian adolescents with long ART experience (31). Inflammatory cytokines such as IL-12 and IFNγ as well as the inflammation-related cytokines IL-6 and IL-1β were lowly expressed among viral failure adolescents, while the expression of inflammatory cytokine CCL3 was higher among those experiencing virological failure. In fact the low level of IFNγ observed in a context of sub-optimal virological response was similar to what have been observed in studies conducted in Brazil and Kenya where a low concentration of IFNγ was observed in adolescents with a detectable viral load (31) and ART non-adherent people living HIV with active viral replication (32). Even though there was not significance association between TNFα and viral load, we have noticed a low level of this inflammation cytokine in adolescents with virological suppression, similarly to what observed in a USA children cohort where low levels of TNFα correlated with low levels of viral load (33). The fact that higher levels of IL-12 was observed in virally suppressed adolescents stresses the immune-stimulatory properties of this cytokine that enhances antiviral activity, thereby contributing in the control of viral replication (34). Furthermore, IL-12 is critical for promoting the differentiation of CD4+ T cells into Th1 cells, which in turn restrains HIV-1 replication and disease progression. As IL-12 is also known to stimulate IFNγ (35), it might be relevant to consider this biomarker in pediatric HIV control or functional cure research strategies. Although IL-10 is generally considered to be an immunosuppressive cytokine that can inhibit antiviral immune responses and promote viral replication (34, 36), we rather observed its expression in a context of viral suppression in this subset of ALPHI and correspondingly with a study conducted in Brazil (31). Thus, IL-10 might have a complex role in HIV pathogenesis, which may be either context- or age-dependent; thereby requiring further investigations to delineate the underpinning mechanism(s).

TNFα and CCL2 are two important cytokines that play a critical role in the immune response during HIV infection. A higher expression level of TNFα was found in immune-competent compared immunocompromised ALPHI, indicating its role in controlling HIV-1 replication (37). In contrast, the high expression of CCL2 in immunocompromised APHI highlights its role in the recruitment of monocytes and other cells to sites of viral replication, leading to possible immune dysfunction (38). Thus, strategies to minimize the expression of CCL2 may strengthen the pediatric immune response, which in turn would support immune normalization and better control of HIV replication.

Looking at the profile of cytokines according to ADRMs, only IL-4 and CCL3 were highly expressed in the presence of ADRMs. To the best of our knowledge, there is currently no evidence to suggest that these biomarkers (IL-4 and CCL3) can influence archiving of HIV-1 drug resistance mutations (39). While calling for further investigations, IL-4 is known to promoting survival of CD4+ T cells (i.e., primary targets for HIV-1 infection) (39), either by impairing HIV-1 replication/transcription in infected cells or by limiting the viral cytopathic effect, which consequently favors viral archiving in cellular reservoirs (40). Inversely, IL-4 may also increase the expression of certain proteins (Tat) involved in HIV-1 replication, thereby enhancing the transcription of viral genes under sub-optimal ART (41). Thus, in the frame of a fully functional ART, exploring the role of IL-4 as a possible adjuvant to sustained viral control may contribute to future off-ART strategies investigational approaches in pediatrics.

## Conclusion

Among ALPHI on ART, evidence of the expression level of cytokines and chemokines shows that TNFα is a specific inflammatory marker contributing to chronic immune activation in the course of pediatric HIV-infection. Regarding the response to ART, IFNγ, IL-1β, IL-6, and IL-12 appear as biomarkers of viral suppression, which highlights their roles as potential adjuvants (in the frame of ART success) toward the development of an optimal pediatric HIV control or functional cure strategy. Furthermore, as CCL2 and CCL3 are associated with virological failure and poor immunity, downregulating these markers may contribute to viral control strategies.

## Data availability statement

The original contributions presented in the study are included in the article/supplementary material. Further inquiries can be directed to the corresponding authors.

## Ethics statement

The studies involving humans were approved by National Ethics Committee for research on human health. The studies were conducted in accordance with the local legislation and institutional requirements. Written informed consent for participation in this study was provided by the participants’ legal guardians/next of kin.

## Author contributions

AJN, JF, and GA conceived the study. ACK, MM, GB, and SD enrolled participants. DT, MM, ON, GB, and SD analyzed samples. ACK, AJN, EN, and ON performed the statistical analysis. JF, C-FP, VC, TN, FC, MS, CN, GH-E, and CT interpreted the data; ACK, JF, AJN, and GA drafted the manuscript. AJN, ADN, EN, NF, MT, SS, DK, FA, NS, AL, TN, ST, AEN, CN, CM, GH-E, CT, VC, MS, FC, C-FP, and JF revised the manuscript. AJN and JF supervised the work. All authors contributed to the article and approved the submitted version.
